# Physical exercise promotes astrocyte coverage of microvessels in a model of chronic cerebral hypoperfusion

**DOI:** 10.1186/s12974-020-01771-y

**Published:** 2020-04-16

**Authors:** Marina Leardini-Tristão, Giulia Andrade, Celina Garcia, Patrícia A. Reis, Millena Lourenço, Emilio T. S. Moreira, Flavia R. S. Lima, Hugo C. Castro-Faria-Neto, Eduardo Tibirica, Vanessa Estato

**Affiliations:** 1 Laboratory of Immunopharmacology, Oswaldo Cruz Foundation, Av. Brasil, 4365, Manguinhos, Rio de Janeiro, 21040-900 Brazil; 2 Laboratory of Cardiovascular Investigation, Oswaldo Cruz Foundation, Rio de Janeiro, Brazil; 3 Laboratory of Glial Cell Biology, Biomedical Sciences Institute, Federal University of Rio de Janeiro, Rio de Janeiro, Brazil; 4 National Institute of Cardiology, Rio de Janeiro, Brazil

**Keywords:** Exercise, Cerebral hypoperfusion, Neuroinflammation, Glial cells, Microcirculation

## Abstract

**Background:**

Brain circulation disorders such as chronic cerebral hypoperfusion have been associated with a decline in cognitive function during the development of dementia. Astrocytes together with microglia participate in the immune response in the CNS and make them potential sentinels in the brain parenchyma. In addition, astrocytes coverage integrity has been related to brain homeostasis. Currently, physical exercise has been proposed as an effective intervention to promote brain function improvement. However, the neuroprotective effects of early physical exercise on the astrocyte communication with the microcirculation and the microglial activation in a chronic cerebral hypoperfusion model are still unclear. The aim of this study was to investigate the impact of early intervention with physical exercise on cognition, brain microcirculatory, and inflammatory parameters in an experimental model of chronic cerebral hypoperfusion induced by permanent bilateral occlusion of the common carotid arteries (2VO).

**Methods:**

Wistar rats aged 12 weeks were randomly divided into four groups: Sham-sedentary group (Sham-Sed), Sham-exercised group (Sham-Ex), 2VO-sedentary group (2VO-Sed), and 2VO-exercised group (2VO-Ex). The early intervention with physical exercise started 3 days after 2VO or Sham surgery during 12 weeks. Then, the brain functional capillary density and endothelial-leukocyte interactions were evaluated by intravital microscopy; cognitive function was evaluated by open-field test; hippocampus postsynaptic density protein 95 and synaptophysin were evaluated by western blotting; astrocytic coverage of the capillaries, microglial activation, and structural capillary density were evaluated by immunohistochemistry.

**Results:**

Early moderate physical exercise was able to normalize functional capillary density and reduce leukocyte rolling in the brain of animals with chronic cerebral hypoperfusion. These effects were accompanied by restore synaptic protein and the improvement of cognitive function. In addition, early moderate exercise improves astrocytes coverage in blood vessels of the cerebral cortex and hippocampus, decreases microglial activation in the hippocampus, and improves structural capillaries in the hippocampus.

**Conclusions:**

Microcirculatory and inflammatory changes in the brain appear to be involved in triggering a cognitive decline in animals with chronic cerebral ischemia. Therefore, early intervention with physical exercise may represent a preventive approach to neurodegeneration caused by chronic cerebral hypoperfusion.

## Background

Dementia is a neurodegenerative disease with a progressive impairment of cognition and behavioral changes that may be preceded or accompanied by loss of neurons, neuroinflammation, and oxidative stress [1, 2]. Brain circulation disorders, such as chronic cerebral hypoperfusion, have been associated with a decline in cognitive function in elderly people and during the development of dementia [3, 4]. Micro- and macrovascular changes are also related to the neurodegenerative process and may occur before the onset of dementia [5]. Vascular dementia highlights the importance of vascular contributions to cognitive impairment and represents the second most common form of dementia following Alzheimer’s disease [6].

In the last few decades, animal models have been used to study alterations and consequences of chronic reduction on cerebral blood flow (CBF) that may occur in neurodegenerative disease. The permanent bilateral occlusion of the common carotid arteries in rats (or 2-vessel occlusion, 2VO) is a cerebral hypoperfusion model that impacts the regions of the brain associated with learning and memory processes, such as the cortex and hippocampus [7]. The chronic phase of the 2VO model, between the eighth and twelfth weeks after the procedure, more closely resembles the decrease in CBF in human aging and dementia. In this phase of the model, the neurodegenerative process is more extensive; therefore, it is the most used phase for the study of dementia [4].

Astrocytes together with microglia participate in the immune response in the CNS. The location of these cells, in or near the blood-brain barrier (BBB), makes them potential sentinels in the brain parenchyma. Astrocytes play an important role in communicating with blood vessels, being involved in the maintenance of the BBB and CBF, providing for the nutrition of neurons, and promoting vasodilation in response to neuronal activation [8]. Microglia are the most common resident immune cells, which represent the first-line of inflammatory response in brain tissue and are generally activated during infections, lesions, or degenerative diseases [9]. Microglia activation in response to injury commonly involves morphological changes leading to transformation from a ramified to amoeboid form [10]. Activation also induces the release of proinflammatory cytokines and chemokines, which promotes the infiltration of circulating leukocytes into the brain, leading to the chronic inflammatory process present in neurodegenerative diseases [11].

Experimental and clinical studies have shown that the acute phase after cerebral ischemia is a critical window for the impairment of neuronal plasticity. Treatment during this period can trigger and promote neuroprotective mechanisms that will assist in the recovery of neuronal functions [12–15]. Among new therapeutic strategies being pursued to minimize cognitive damage, physical exercise has been shown to support brain health and function, with an impact on neurogenesis [16, 17], reducing neuroinflamation [14, 18] and oxidative stress [14, 19]. Moreover, regular physical exercise is able to improve cognitive function in animals [20, 21], and in adult and elderly populations [22–24].

This paper describes a new approach to the neuroprotective role of physical exercise in chronic hypoperfusion, a common feature of dementia. It is possible that exercise modulates the interaction between glial cells and brain microcirculation, but this hypothesis has not yet been fully investigated. Thus, using the 2VO model, we investigated the impact of early moderate exercise as a therapeutic strategy on brain microcirculation, neuroinflammation, and astrocytic coverage of brain vessels.

## Methods

### Animals and ethics statement

Fifty-eight male Wistar rats (Oswaldo Cruz Foundation Animal Facilities, Rio de Janeiro, Brazil), 12 weeks old, were housed under controlled temperature (21 ± 2 °C) and light (12 h light/dark cycles) conditions and were allowed free access to water and standard rat chow. All experiments were conducted in accordance with internationally accepted principles for the care and use of laboratory animals and were approved by the Animal Ethics Committee of the Oswaldo Cruz Foundation (protocol number L-002/2016).

### Experimental design

We analyzed the effects of physical exercise on cerebral hypoperfusion in the two-vessel occlusion (2VO) experimental model. We chose the cerebral hypoperfusion model because it is a validated and representative model of changes in the cerebral blood flow of aging without, therefore, including the bias of the physical limitations of aging in the performance of physical exercise [4]. Animals were randomly assigned to the following four experimental groups: (1) subjected to cerebral hypoperfusion and exercise (2VO-Ex; *n* = 15); (2) subjected to cerebral hypoperfusion and non-exercised (sedentary) (2VO-Sed; *n* = 15); (3) subjected a sham procedure and exercise (Sham-Ex; *n* = 12); and (4) subjected a sham procedure and non-exercised (sedentary) (Sham-Sed; *n* = 11). The animals in the sham groups were subjected to the same surgical procedure without ligation of the left and right common carotid arteries.

Three days after surgery, the exercised groups performed maximal exercise testing (MET). The exercise protocol then started 24 h following MET. After completing 12 weeks of a moderate physical exercise protocol, the animals underwent cognitive, hemodynamic, microcirculatory, and inflammatory assessments.

### Two-vessel occlusion (2VO) surgical procedure

In order to promote a global and chronic cerebral hypoperfusion, rats were anesthetized intraperitoneally (i.p.) with ketamine (90 mg/kg) and xylazine (10 mg/kg), and underwent 2VO surgery as previously described (Farkas et al. 2004). Briefly, a ventral incision was made in the midline of the neck using a scalpel to expose the left and right common carotid arteries, which were gently separated from the vagus nerve. Animals in the 2VO group had both vessels permanently occluded with 6-0 silk suture. The sham groups were subjected to the same procedure without occlusion of the carotid arteries. After surgery, the animals received subcutaneous injections of an anti-inflammatory drug, ketoprofen (1 mg/kg/day for 3 days) and an antibiotic, meropenem (10 mg/kg, single dose). The animals were kept in individual cages until the end of the experimental protocol to avoid stress [14, 25].

### Aerobic exercise training protocol

Before surgery, all the animals were adapted to a treadmill for rats (Model HT 2.0 Hectron Fitness Equipment, Rio de Janeiro, Brazil) by running at a speed of 10 m/min for 10 min over a period of 3 days. Then, the 3 days after surgery the MET was performed to establish the appropriate training intensity [26]. The MET began at a speed of 10 m/min; the speed was then increased by 3 m/min every 3 min until the animals reached exhaustion and remained on the shock grid for more than 5 s. For each MET trial, time to exhaustion, maximal speed, and maximal distance were recorded. Maximal speed was used to determine the training intensity for the duration of the investigation, which was 60% of the MET. The MET was also performed on the sixth week of exercise to adjust the intensity of the protocol.

The physical exercise consisted of 30-min sessions, 3 times a week over 12 weeks at 60% of the maximal speed, which occurred between 8:00 and 10:00 am. Twenty-four hours after the last exercise for the exercised group or sedentarism for the sedentary group, the animals underwent additional procedures for cognitive function, hemodynamic parameter levels, microcirculatory, and neuroinflammatory alterations.

### Hemodynamic assessment

In order to monitor blood pressure, systolic blood pressure (SBP) was assessed in conscious animals one day before 2VO/sham surgery (baseline measurement), 48 h after 2VO/sham surgery and after 12 weeks of exercise or sedentarism using a computerized tail-cuff plethysmography system (BP-2000; Visitech Blood Pressure Analysis System, Apex, NC, USA). The animals were adapted to the apparatus for three consecutive days prior to undergoing their baseline measurements.

### Open-field test

At the end of 12 weeks of exercise, cognitive function was investigated by an open-field test. Habituation to an open field was carried out as described by Vianna and coworkers [27], and consists of quantitatively assessing the exploratory and locomotor activities, as well as the habituation memory of the animals. Briefly, animals were gently placed in the center of an open box (50 cm high, 50 cm wide, 39 cm deep) with the floor divided into a grid and allowed to explore the arena for 5 min (training session). After 24 h the animals were submitted to a similar open-field session (test session). Crossing of the gridlines and rearing (rearing up on the hind legs) performed in both sessions were counted.

### Intravital microscopy imaging

Brain microcirculation of all groups of animals was observed via cranial window imaging, and the functional capillary density and leukocyte-endothelium interactions were evaluated by fluorescence intravital video microscopy as previously described [28, 29]. Briefly, the animals were anesthetized with ketamine (90 mg/kg, i.p.) and xylazine (10 mg/kg, i.p.) and fixed in a stereotaxic frame. Then, their left parietal bones were exposed via midline skin incisions, and a cranial window on the right parietal bone (5 mm diameter, between the coronal and lambdoid sutures) was created with a high-speed drill. The dura mater and arachnoid membranes were subsequently excised and withdrawn to expose the cerebral microcirculation, after which the cranial windows were suffused with artificial cerebrospinal fluid (132 mM NaCl, 2.95 mM KCl, 1.71 mM CaCl_2_, 0.64 mM MgCl_2_, 24.6 mM NaHCO_3_, 3.71 mM dextrose, and 6.7 mM urea, pH 7.4, at 37 °C).

The visualization of the brain microvascular surface was facilitated by intravenous administration of 0.1 mL of 2% fluorescein isothiocyanate (FITC)-labeled dextran using a microscope and epi-illumination (Olympus BX150WI, NY, USA) outfitted with a fluorescent light source (460 to 490 nm using a 520 nm emission filter). Microscopic images were subsequently acquired, and capillaries were counted using Cell Sens Standard 1.9 software. The functional capillary density, defined as the total number of spontaneously perfused capillaries per square millimeter of surface area, was determined in the random microscopic field of view over a 4-min period [29].

Leukocytes were fluorescently labeled by intravenous administration of rhodamine 6G (0.3 mg/kg) and observed through the cranial window. Adherent leukocytes were defined as the number of leukocytes adhered to the venular endothelium for a period of 30 s or longer and expressed as cell number/min/100 μm. Leukocytes were considered rolling on the vessel wall if they moved at a slower rate than circulating erythrocytes, and expressed in cells/min. Brain surface venules with diameters ranging from 50 to 100 μm were analyzed [29].

### Tissue processing

After intravital microscopy, rats were euthanized by pentobarbital overdose (150 mg/kg, i.p.). The brain was dissected, and the hippocampus and left hemisphere cortex regions were stored in the freezer at – 80 °C for subsequent western blot analysis. For immunohistochemical analysis, 5 animals per group were anesthetized with ketamine and xylazine (90 and 10 mg/kg i.p., respectively) and transcardially perfused with 0.9% saline followed by 4% paraformaldehyde and the brains were then removed and maintained in paraformaldehyde solution.

### Western blot analysis

Protein levels of synaptophysin and postsynaptic density protein 95 (PSD-95) in the hippocampus were determined by western blot analysis. The hippocampus was homogenized in 50 mM Tris, pH 8.0, containing 150 mM NaCl, 1 mM EDTA, 1% Triton X-100, 0.5% sodium deoxycholate, 0.1% SDS, and protease inhibitor cocktail (Complete Mini Protease Inhibitor Cocktail; Roche Diagnostics, IN, USA). Homogenates were incubated for 30 min on ice, sonicated for 3 min, and centrifuged at 10000×*g* at 4 °C. The supernatant was collected, and the protein concentration was measured using bicinchoninic acid method, with bovine serum albumin (BSA) as the standard (BCA Protein Assay Kit, Thermo Scientific, Waltham, MA, USA). Then, 50 μg of protein per lane was separated on a 12% SDS-PAGE gel and transferred to a nitrocellulose membrane (BioRad Laboratories, Munich, Germany). After blocking with 5% BSA (Sigma Aldrich, St. Louis, MO, USA), the membranes were incubated overnight at 4 °C with antibodies to synaptophysin and PSD-95 (1:500 and 1:5000 respectively; Abcam) followed by a secondary antibody (1:20000). Beta actin (1:10000; Abcam) was used as a loading control. The data were obtained by Odyssey software (Li-Cor Biosciences, Lincoln, NE, USA).

### Immunohistochemistry

For immunohistochemistry analysis, brains were quickly excised after perfusion-fixation as described above and serially sectioned at 50 μm. The sections were washed with PBS and incubated with 10% NGS diluted in PBS with 0.3% Triton X-100 for 90 min. They were then incubated with antibodies to the glial fibrillary acidic protein (GFAP, Dako; 1:400) and the ionized calcium-binding adaptor molecule 1 (Iba1, Wako; 1:200) or with biotinylated IB4 (Vector; 1:100) overnight at 4 °C, then washed again with PBS and incubated with secondary antibodies conjugated with Alexa Fluor 488 or 546 (1:400) or streptavidin-Cy3 (1:400) for 2 h. The sections were counterstained with DAPI and coverslips were mounted with fluoromount. Negative controls were performed with non-immune rabbit IgG. Slices were imaged using a confocal microscope (Leica TCS-SP5) equipped with a 63X NA 1.40 oil-immersion objective. We were careful to apply the same selection criteria to all cuts. Initially, we located the CA1 region of the hippocampus and then we located the parietal cortex. Image processing was performed using Fiji, ImageJ software.

For microglial morphology analysis, the ImageJ Sholl Analysis tool was used. This is a quantitative analysis method commonly used in neuronal studies to characterize the morphological characteristics of an imaged neuron. Using Sholl analysis, a mathematical algorithm of the program called “branching index” is used to analyze neuronal morphology. This index compares the difference in the number of intersections make consecutive circles of the Sholl analysis in relation to the distance of the neuronal sum. In this study, microglial processes were quantified as a number of intersections from 2 to 40 μm from the sum and total number of intersections within the analysis circles, representing the total branch of the microglia.

For the analysis of the cerebral microvascular angioarchitecture, the AngioTool program was used (https://ccrod.cancer.gov/confluence/display/ROB2/Downloads) to determine the total length of the brain capillaries on each image obtained by confocal microscopy. AngioTool is a validated source for measuring vascular networks [30] and has already been described in analyses of murine brain and retinal angiogenesis [31, 32].

### Statistical analysis

The results are expressed as the mean ± standard error of the mean (SEM) for each group. Between-group statistical comparisons were performed using one-way analysis of variance (ANOVA), followed by Bonferroni’s post hoc test. When appropriate, the results were analyzed using unpaired Student’s *t* test*.* MET and open-field parameters were analyzed using paired Student’s *t* tests. Systolic blood pressure before and 48 h after surgery were analyzed using Student’s *t* tests*.* Differences with *p* values less than 0.05 were considered significant. All calculations were performed using commercially available statistical software (GraphPad Prism Software, CA, USA).

## Results

### Early moderate physical exercise reduced systolic blood pressure in animals with chronic cerebral hypoperfusion

The SBP of all animal groups was assessed before 2VO or sham surgery, 48h after 2VO or sham surgery and at the end of 12 weeks of physical exercise (2VO-Ex and Sham-Ex) or physical inactivity (2VO-Sed and Sham-Sed). At baseline, all animals exhibited similar SBP (data not shown). As expected, 48 h after 2VO surgery an increase in SBP was observed, compared to those submitted to sham surgery (Table 1). At 12 weeks the SBP was higher in the 2VO-Sed group compared to the respective sham groups. Moreover, physical exercise was able to bring SBP back to basal levels in the 2VO-Ex group.

**Table 1:** Systolic blood pressure (SBP) during chronic cerebral hypoperfusion

	Groups	SBP
48 h after surgery	Sham	130.4 ± 2.24
	2VO	147 ± 4.30 ^§§^
after 12 weeks with or without physical exercise	Sham-Sed	131 ± 2.3
	Sham-Ex	125 ± 4.44
	2VO-Sed	140.7 ± 1.75*
	2VO-Ex	130 ± 2.06 #

Systolic blood pressure (SBP) during chronic cerebral hypoperfusion. Results are expressed as the mean ± SEM of 9 animals per group. SBP at 48 h after sham or 2VO surgery. Systolic blood pressure before and 48 h after surgery were analyzed using Student’s *t* tests and after 12 weeks with or without physical exercise was analyzed using ANOVA*. Sham* sham surgery group, *2VO* chronic cerebral hypoperfusion, *Sham-Sed* sham surgery non-exercised group, *Sham-Ex* sham surgery exercised group, 2*VO-Sed* chronic cerebral hypoperfusion non-exercised group, *2VO-Ex* chronic cerebral hypoperfusion exercised group

^§§^*p*<0.01 vs. Sham

^#^*p* < 0.05 vs. 2VO-Sed

^##^*p* < 0.01 vs. 2VO-Sed

### Physical performance was not altered in animals with chronic cerebral hypoperfusion

Prior to the physical exercise protocol, animals were adapted to the treadmill for 3 days before the surgical procedure, and then submitted to the MET for the correct prescription of exercise intensity for the course of this investigation. Table 2 shows that both exercised groups presented improvement at the readjustment MET when compared with pre-training values, regarding time to exhaustion, maximal speed, maximal distance and training velocity. No differences were found between the groups that received either the 2VO or sham surgery.

**Table 2:** Physical performance in the maximal exercise test

Parameter	Sham-Ex	2VO-Ex
Time to exhaustion (min)		
Baseline	14.52 ± 1.74	14.63 ± 2.14
Readjustment	20.89 ± 1.21 **	19.57 ± 1.89^§^
Maximal speed (m/min)		
Baseline	25.00 ± 0.91	25.75 ± 0.84
Readjustment	29.10 ± 1.23 *	30.00 ± 0.77^§^
Maximal distance (m)		
Baseline	283.00 ± 23.38	322.00 ± 23.64
Readjustment	405.00 ± 31.64*	429.70 ± 23.03^§§^
Training velocity (m/min)		
Baseline	15.00 ± 0.54	15.45 ± 0.50
Readjustment	17.45 ± 0.74 *	18.00 ± 0.46^§§^

MET was performed 3 days after 2VO or sham surgery and on the sixth week of exercise to determine the appropriate exercise training for the duration of the investigation. Sham-Ex, sham surgery exercised group (*n* = 12); 2VO-Ex, chronic cerebral hypoperfusion exercised group (*n* = 15). Values represent the mean ± SEM

*p < 0.05 vs. Sham-Ex baseline

**p < 0.01 vs. Sham-Ex baseline

^§^*p* < 0.05 vs. 2VO-Ex baseline

^§§^*p* < 0.01 vs. 2VO-Ex baseline (Student’s *t* test)

### Early moderate physical exercise was able to improve memory habituation in animals with chronic cerebral hypoperfusion

**Fig. 1:**
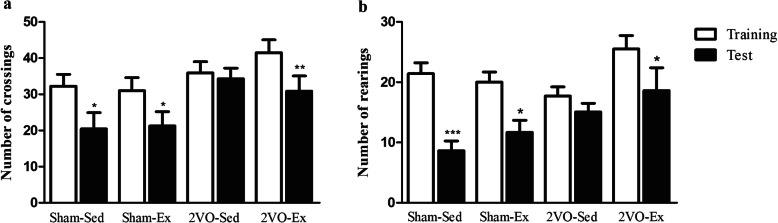
Analysis of cognitive function by open-field test. Bars represent the mean ± S.E.M. (*n* = 12–15 animals per group). **a** The number of gridline crossings, and **b** the number of hind leg rearing of the animals on the training day (white bars) and 24 h later on the test day (black bars). Sham-Sed, sham surgery non-exercised group; Sham-Ex, sham surgery exercised group; 2VO-Sed, chronic cerebral hypoperfusion non-exercised group; 2VO-Ex, chronic cerebral hypoperfusion exercised group. *p < 0.05, **p < 0.01, and ***p < 0.001 vs. training day (paired Student’s *t* test)

After 12 weeks of physical exercise or sedentarism, animals were evaluated for cognitive function by the open-field test. This test is based on the tendency of rodents to explore a new environment more than a familiar one. During a training day, animals are allowed to explore and familiarize themselves with an open-field arena, which has a grid marked. The following test day, the animals are expected to recognize and explore the environment less. For the Sham-Sed and Sham-Ex groups, there were a reduced number of gridline crossings and hind leg rearings in the test day suggesting familiarity with the environment (Fig. 1a, b). Whereas the 2VO-Sed animals showed no difference in the exploratory profile on training and test days. This trend was absent in the 2VO-Ex animals which demonstrated a reduction in environmental exploration on the test day indicating intact cognitive skills.

### Early moderate physical exercise was able to normalize functional capillary density and reduce leukocyte rolling in the brain of animals with chronic cerebral hypoperfusion

**Fig. 2:**
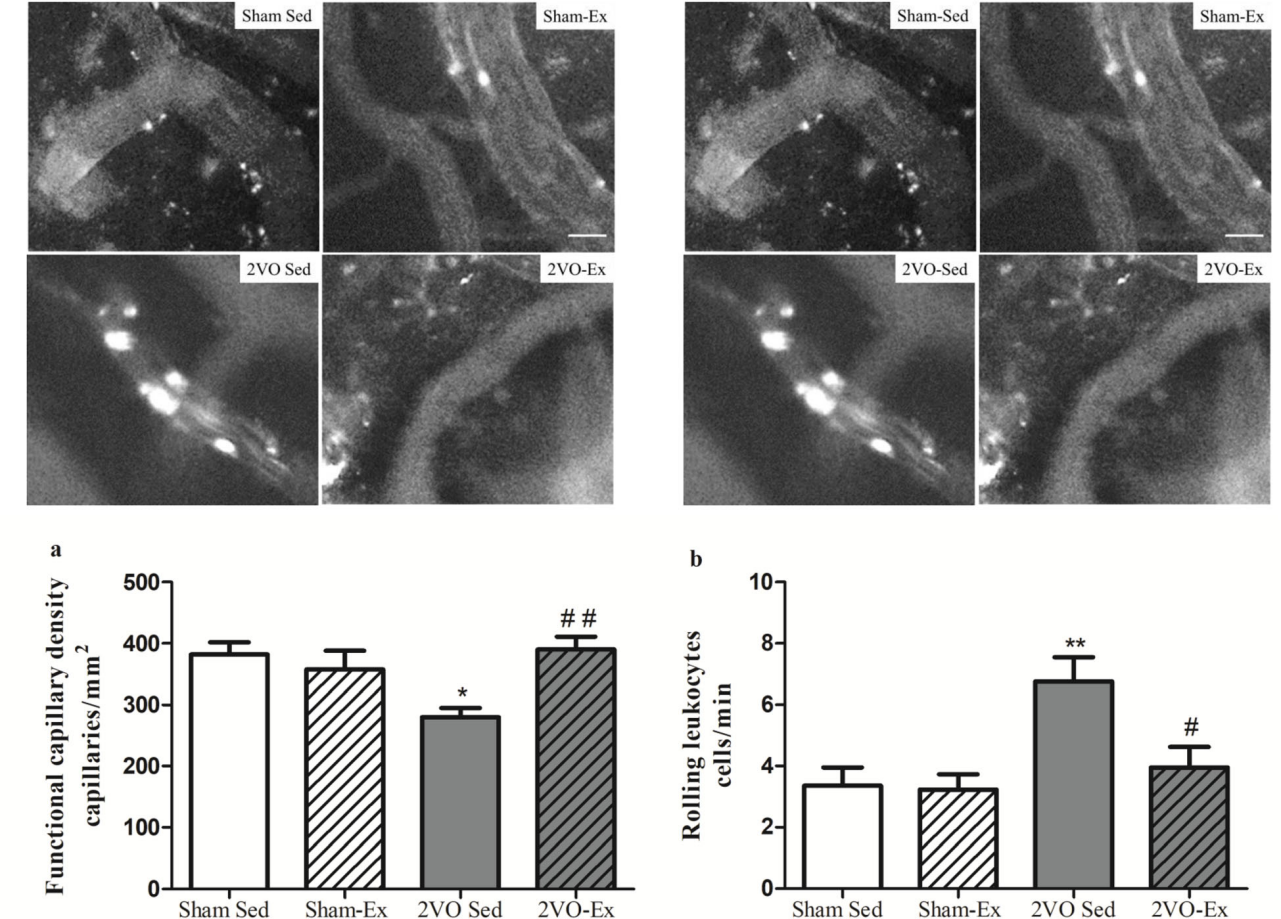
Representative images of cerebral intravital microscopy and microcirculation analysis of the cerebral cortex. The values represent the mean ± S.E.M (*n* = 6–8 per group). Bar graphs represent **a** the functional capillary density, and **b** the number of rolling leukocytes in venules after 12 weeks of physical exercise or sedentarism. Sham-Sed, sham surgery non-exercised group; Sham-Ex, sham surgery exercised group; 2VO-Sed, chronic cerebral hypoperfusion non-exercised group; 2VO-Ex, chronic cerebral hypoperfusion exercised group. In **a** *p < 0.05 vs. Sham-Sed and ^##^*p* < 0.01 vs. 2VO-Sed (unpaired Student’s *t* test); in **b** **p < 0.01 vs. Sham-Sed and ^#^*p* < 0.05 vs. 2VO-Sed (ANOVA). Scale bar 100μm, magnification100X in a and 200X in b

We next analyzed brain microcirculation and inflammatory cell dynamics by intravital microscopy. A reduced number of perfused capillaries was observed in the cortical area of the brains of the 2VO-Sed group when compared to Sham-Sed group (Fig. 2a). The reduction in the number of perfused capillaries was prevented in the 2VO-Ex group, as capillary density in the 2VO-Ex was not significantly different from the sham groups.

We also evaluated the rolling of leukocytes on postcapillary venules. Changes in leukocyte-endothelial interactions were observed in the 2VO-Sed animals, which exhibited increased leukocyte rolling in brain vessels when compared to the Sham-Sed group, while physical exercise prevented this inflammatory event in the 2VO-Ex group (Fig. 2b).

### Early moderate physical exercise restored synaptic proteins expression in the brain of animals with chronic cerebral hypoperfusion

**Fig. 3:**
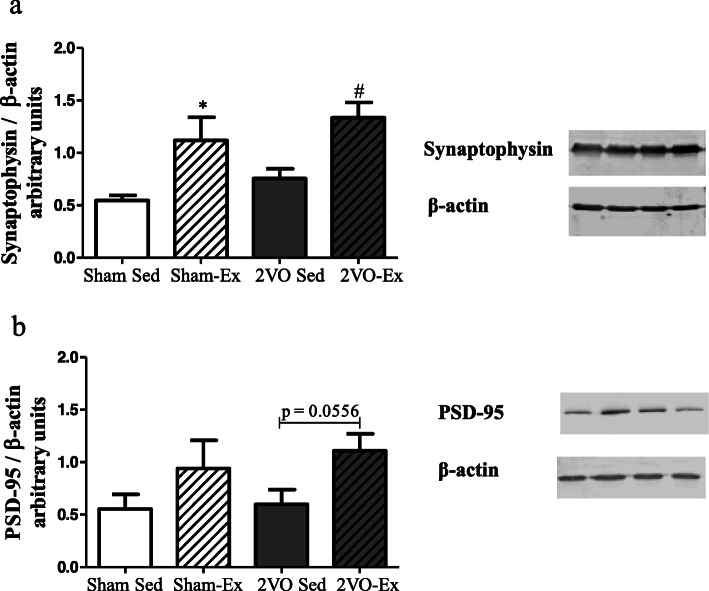
Western blot analysis for the expression of synaptophysin (**a**) and PSD-95 (**b**) in the hippocampus. Values represent the mean ± S.E.M. of 4–7 animals per group. Quantitative protein expression of synaptophysin and PSD-95 bands detected by Western blot using β-actin as a loading control (**c**). Sham-Sed, sham surgery non-exercised group; Sham-Ex, sham surgery exercised group; 2VO-Sed, chronic cerebral hypoperfusion non-exercised group; 2VO-Ex, chronic cerebral hypoperfusion exercised group. In **a** *p < 0.05 vs. Sham-Sed and ^#^*p* < 0.05 vs. 2VO-Sed (unpaired Student’s *t* test); in **b** #*p* = 0.0556 vs. 2VO-Sed (unpaired Student’s *t* test).

In order to investigate the effects of chronic hypoperfusion on the expression of hippocampal plasticity-related proteins, we performed western blots for the detection of synaptophysin and PSD-95 in the hippocampus of animals. Early physical exercise was able to increase synaptophysin expression in the brain of both exercised groups (Fig. 3a). Similarly, increased expression of PSD-95 was detected in the 2VO-Ex group with statistical significance of *p* = 0.556 (Fig. 3b).

### Early moderate physical exercise improves astrocytes coverage in blood vessels of the cerebral cortex and hippocampus of animals with chronic cerebral hypoperfusion

**Fig. 4:**
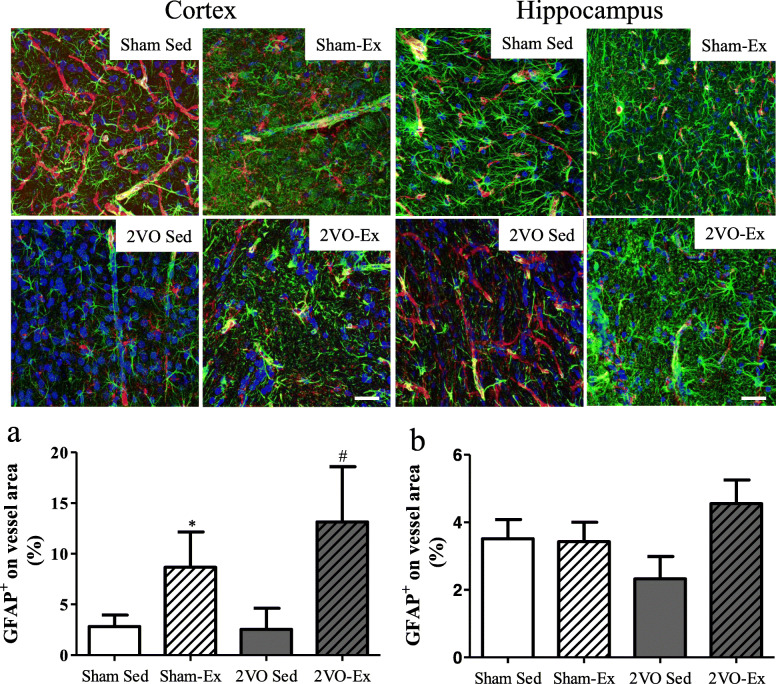
Immunohistochemical assessment of vessels covered by astrocytes in the cerebral parietal cortex and in the CA1 region of hippocampus. Representative confocal images of astrocyte coverage (GFAP, green) in vessels of the cerebral cortex and hippocampus (IB4, red) of animals which were exercised or sedentary for 12 weeks after cerebral hypoperfusion (2VO-Ex and 2VO-Sed, respectively) or after sham surgery (Sham-Ex and Sham-Sed, respectively). DAPI-labeled nuclei, blue. Quantification of the **a** cerebral cortex and **b** hippocampus. Data represent the mean ± SEM of up to 10 vessels per image of 3-4 animals per group. *p < 0.05 vs. Sham-Sed and ^#^*p* < 0.05 vs. 2VO-Sed (ANOVA). Scale bar 50 μm, magnification 200X

We also investigated the interaction of astrocytes with the vasculature in the cortex and hippocampus (Fig. 4a). Immunohistochemical analysis allowed the evaluation of vessel coverage by astrocytes on the cerebral microcirculation, which is an important feature of the neurovascular unit and BBB integrity. Animals that were submitted to chronic cerebral hypoperfusion but not to physical exercise presented a significant reduction in the amount of IB4^+^ vessels surrounded by GFAP^+^ astrocytes in the cortex and hippocampus (Fig. 4). Physical exercise recovered astrocytes vessel coverage in both the cortex and hippocampus in the 2VO animals (Fig. 4a, b). Although there were no differences between the Sham-Sed and Sham-Ex groups compared to the 2VO-Ex in the hippocampus, there was a reduction in the Sham-Sed group in the cerebral cortex.

### Physical exercise decreases microglial activation in the hippocampus from animals submitted to chronic cerebral hypoperfusion

**Fig. 5:**
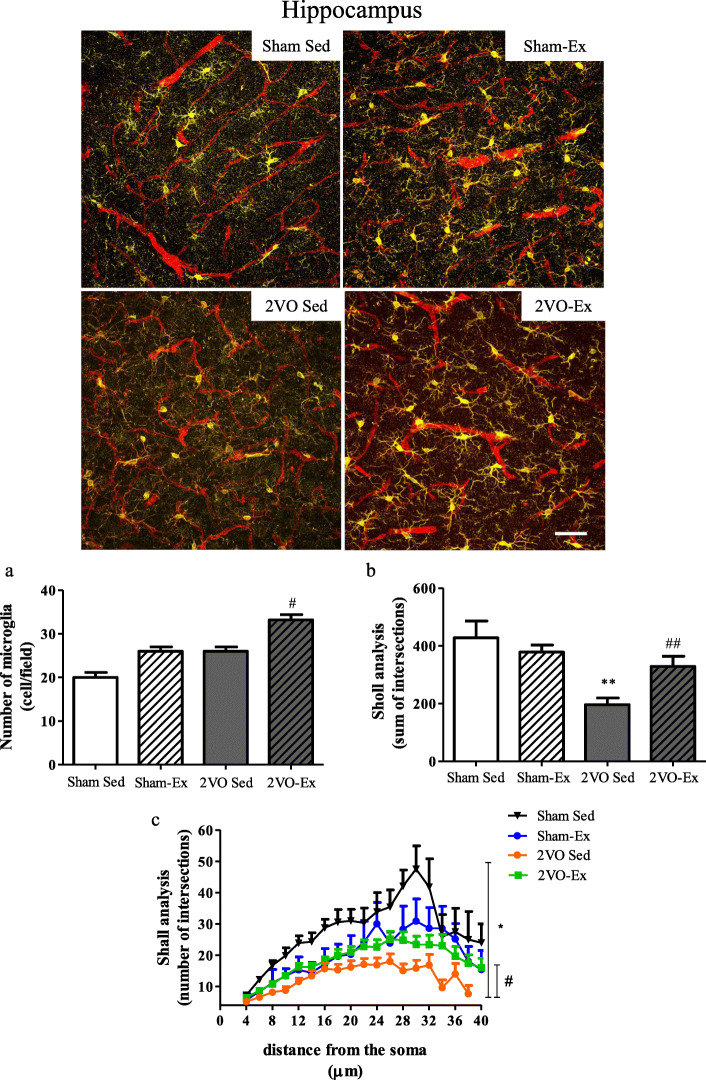
Exercise increases the number of microglia process in the CA1 region of hippocampus following cerebral hypoperfusion. Representative confocal images of microglia morphology (Iba1^+^, yellow) in vessels of the hippocampus (IB4, red) of animals that were exercised or sedentary for 12 weeks after cerebral hypoperfusion (2VO-Ex and 2VO-Sed, respectively) or after sham surgery (Sham-Ex and Sham-Sed, respectively). Data represent the mean ± SEM of up to 40 microglial cells per group of 3 animals. In **a** number of microglia in the hippocampus, **b** and **c** number of microglia branches. *p < 0.05 vs. Sham-Sed and **p < 0.01 vs. Sham-Sed; ^#^*p* < 0.05 and ^##^*p* < 0.01 vs. 2VO-Sed (ANOVA). Scale bar 50 μm, magnification 200X

We next investigated the effects of physical exercise on microglial morphology following the 2VO protocol. Although isolectin IB4 stains both microglia and vessels, labeling with the microglia-specific IBA1 enabled the morphology of these cells to be observed more clearly. Analysis of parenchymal Iba1^+^ cells in the 2VO-Ex group revealed an increase in the number of microglia compared to respective sedentary group (Fig. 5a). In the 2VO-Sed group, microglia displayed an amoeboid phenotype and increased soma when compared to Sham-Sed group, whilst both the 2VO-Ex and the Sham-Ex groups presented microglia with more ramified morphology (Fig. 5b). This was supported by quantitative data obtained by Sholl analysis showing that the maximum number of microglia process interactions and the distance from the soma where these interactions occurred was significantly decreased in the 2VO-Sed group, but not in the exercised groups, that had levels similar to sham animals (Fig. 5c).

### Early moderate physical exercise improves structural capillaries in hippocampus

**Fig. 6:**
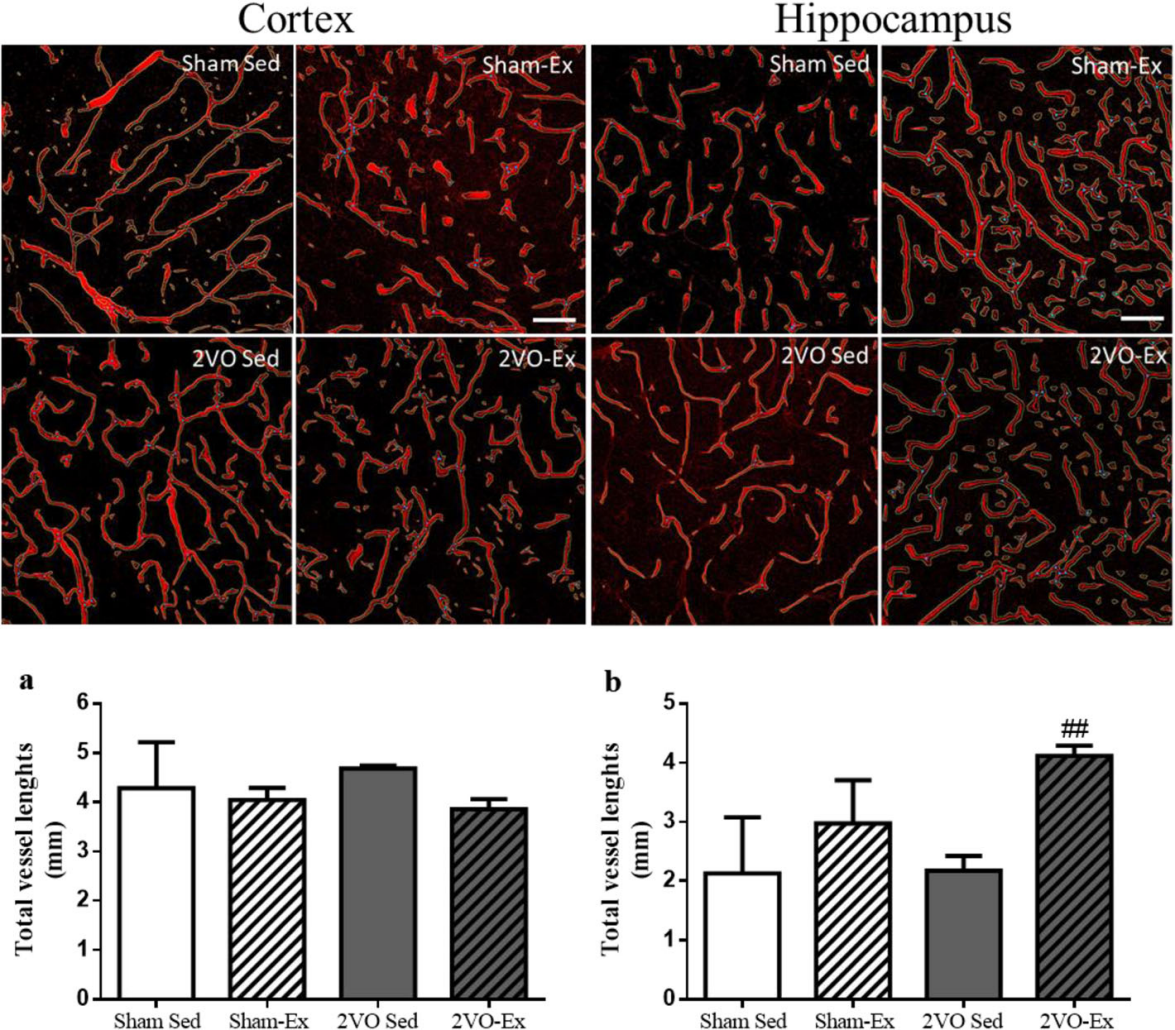
Structural brain capillaries assessed by AngioTool analysis. Data represent the mean ± SEM of 4 images per group of 3 animals. Total length of the vessels in **a** the cortex and **b** the hippocampus of rats submitted to 2VO and sedentary (2VO-Sed) or exercised (2VO-Ex) for 12 weeks, and their respective controls that had been submitted to sham surgery (Sham-Sed and Sham-Ex). ^##^*p* < 0.01 vs. 2VO-Sed (ANOVA).Scale bar 50 μm, magnification 200X

Further investigation of the structural capillaries in the cortex and hippocampus was performed by the AngioTool technique. There were no significant differences in the length of the capillaries of the cortex (Fig. 6a). However, exercise was able to increase capillary length in the hippocampus in the 2VO-Ex group compared to the sedentary 2VO-Sed group (Fig. 6b).

## Discussion

Using a two-vessel occlusion model, 2VO, in rats we found that chronic cerebral hypoperfusion (i) caused a reduction in habituation memory; (ii) induced brain functional capillary rarefaction, as well as an increase in rolling leukocytes to the microvascular endothelium; (iii) induced alterations in astrocyte coverage of brain microvessel; and (iv) induced microglia activation phenotype. In addition, early intervention with moderate physical exercise over a period of 12 weeks effectively reduced systolic blood pressure and vascular inflammation in the post-capillary venules; increased the total length of capillaries in the hippocampus; improved coverage of astrocytes on cerebral capillaries; improved microglia activation phenotype; and improved habituation memory accompanied by an increase in the expression of synaptophysin and PSD-95 proteins.

Following the 2VO surgery, the systolic blood pressure was increased, which likely occurred due to a reduction in carotid blood flow after occlusion. This in turn reduces the stretching of the arterial walls resulting in reduced baroreceptor stimulation, which responds to the fall in blood pressure by increasing sympathetic tone, consequently increasing the systemic blood pressure [4]. Previous studies that analyzed the mean blood pressure found significant increases after 2VO in both the short-term [14, 33] and the long-term [34]. This was reported by Farkas and colleagues who described a 10 to 20 mmHg increase in systolic blood pressure at 24 h after 2VO, persisting for up to 9 weeks [4]. Physical training, particularly aerobic exercise, has been shown to be an effective non-pharmacological therapeutic approach in the reduction of blood pressure in models of metabolic syndrome [35] and hypertension [36]. A normalization in systolic blood pressure was observed in our 2VO model after 12 weeks of moderate exercise compared to animals that were sedentary for this period. Endothelial dysfunction is considered one of the central mechanisms of structural and functional vascular changes in neurodegenerative diseases. In the 2VO model, it has been observed that endothelial activation markers increase 1 day post-occlusion to 28 days, with a peak at 3 days [37]. Yata et al. found increased leukocyte rolling and adhesion in pial surface venules after 24 h, 1, and 2 weeks of cerebral hypoperfusion suggesting that leukocyte activation may be the first step in the whole neurovascular unit stress response to chronic cerebral hypoperfusion [38]. Previous results from our group have shown that physical exercise initiated early following 2VO and maintained for 1 week was able to reduce adherent leukocytes in brain venules and to reduce NADPH-oxidase expression in the brain [14]. Here, we observed that 12 weeks of physical exercise was able to reduce the rolling of leukocytes on brain venules.

Further positive effects of physical exercise following 2VO were observed, such as the ability to normalize the functional capillary density in the cerebral cortex and an increase in the total length capillaries in the hippocampus. Our observations are in agreement with previous studies showing that physical training can induce an increase in cortical capillary density and lead to the growth of new capillaries in the brain, especially in the motor cortex [39–41]. Although we have not quantified the influence of angiogenic factors on the response of physical exercise to cerebral microcirculation, several studies suggest the participation of VEGF as an important factor in increasing the structural capillary density in central and peripheral vascular beds after physical exercise [42, 43]. Physical exercise for 4 months in aged rats was able to increase the volume, capillary length, and total surface area of capillaries in the cerebral cortex [44]. De Jong et al. observed that the cognitive performance of rats submitted to 2VO correlated closely with capillary morphology in the hippocampal CA1 area, suggesting that capillary integrity is one of the important determinants of brain function [45]. Furthermore, a positive correlation between cognition and brain nutrition has been demonstrated in several studies [45, 46].

Our results showed that when animals are sedentary following chronic cerebral hypoperfusion their exploratory response was unchanged when presented a second time to a same environment, suggesting they did not consolidate memory. Cognitive dysfunction analyzed by other tests, such as the Morris water maze or radial arm maze tasks, has been widely reported in this model during the chronic phase [47, 48]. However, upon physical exercise the animals explored the environment less in the test session when compared to the training session, suggesting familiarity with the environment and thus normal cognitive function. A study by Choi et al. corroborates these findings by showing that aerobic exercise, started 3 weeks after 2VO and maintained for 4 weeks, was able to reduce cognitive impairment and increase hippocampal neurogenesis in a murine model of dementia [49]. Cechetti et al. also demonstrated that forced treadmill exercise prevents oxidative stress and memory deficits following chronic cerebral hypoperfusion [50].

Although the exact molecular mechanisms by which physical training affects brain function are unclear, it is suggested that inhibition of cellular and molecular pathways involved in neuroinflammation contributes to neuroprotection. Positive regulation of the expression of neurotrophic factors, such as synaptophysin, a presynaptic marker, and PSD-95, a postsynaptic marker may help in understanding the protective effects induced by physical exercise. We analyzed expression of these proteins in the hippocampus, which is the memory storage center and an important region for the habituation memory to a new environment. We observed that moderate physical exercise for 12 weeks was able to increase protein content of synaptophysin and PSD-95 in animals submitted to 2VO model. Although not significant, the decrease in blood pressure observed in the Sham-Ex group may be related to the increase in synaptic proteins synaptophysin, therefore, it can contribute to the improvement of neuronal plasticity, even in the absence of cerebral hypoperfusion. Corroborating our results, other experimental studies have also shown that aerobic exercise was able to increase synaptophysin and PSD-95 levels in the hippocampus and prefrontal cortex of healthy, ischemic, and obese rats [51–53].

Microvascular inflammation and endothelial activation markers are associated with progressive changes in neurovascular unit. It is still unknown whether inflammation is the main cause of the cognitive impact in cerebrovascular diseases and whether this is triggered by cerebral or systemic processes. However, interruption of the interaction between glia cells and vessels probably contributes to the disruption of homeostasis [54].

Microglia are CNS-resident immune cells that actively participate in neuronal homeostasis and adult neural pathology. Under normal physiological conditions, the microglia monitors the microenvironment and communicates bidirectionally with the neurons [55]. During their native resting state, microglia have small cell bodies with numerous long and highly branching processes, displaying high ramification. However, during pathological conditions such as infections, trauma, or cerebral ischemia, microglia rapidly change their morphology from branched cells to reactive cells with thickened and retracted processes, acquiring a more rounded shape, referred to as amoeboid [55, 56]. Chronic microglial activation is an important feature of many neurodegenerative diseases contributing significantly to disease progression [10]. Thus, the modulation of microglial activation has been the subject of intensive research efforts as a potential therapeutic target.

Physical exercise as a therapeutic approach has been shown to significantly attenuate microglia activation in aging [57–59]. The improvement in cognitive function was associated with changes in modulation of the microglial phenotype from proinflammatory M1 to anti-inflammatory M2 in the corpus callosum, after 28 days of exercise in rats with chronic cerebral hypoperfusion [60]. These studies support our data showing that moderate physical training reduced microglial activation and increased the number of its branches in rats with brain chronic hypoperfusion. Branched microglia are constantly “monitoring” the microenvironment in order to respond promptly to a pathological event. Results from other groups indicated that exercise for 8 weeks before the 2VO was able prevent the activation of microglia and astrocytes, accompanied by reduction of neuronal apoptosis [61].

In addition to microglial analysis, we examined the protective effects of moderate physical exercise on vessel coverage by astrocytes in animals with chronic cerebral hypoperfusion. These cells are part of the neurovascular unit and BBB, playing a key role in the structure of the BBB, in the communication with neurons, glutamate homeostasis [62], and also stimulating the release of vasoactive compounds in microcirculation.

Previous experimental work has shown that physical exercise was able to increase the levels of the astrocyte marker, GFAP, in the hippocampus [63] and induce changes in the astrocyte morphology, suggesting beneficial effects on neuronal activity and plasticity [63]. In a recent study, an increase in the number of astrocytic processes during the exercise protocol was observed in other brain areas, such as the prefrontal cortex, striatum, and entorhinal cortex of mice [64].

In the present work, we used immunohistochemistry analysis to evaluate the coverage of astrocytes on brain microvessels. Our results showed that early and long-term physical exercise after cerebral hypoperfusion increased the astrocytic coverage of cerebral blood vessels in parietal cortex and CA1 region of the hippocampus, regions of the brain associated with learning and memory processes. Astrocyte coverage is important for BBB integrity and for the cross-talk between neurons and vessels. This result highlights the important impact of physical exercise on the neurovascular unit.

Until recently, reactive astrogliosis was a reliable and sensitive marker of diseased tissue. However, there is a growing body of evidence that points towards the potential protective contributions of astrocytes during to pathological mechanisms. Astrocytes are considered to provide a suitable environment for neuron function. It is known that the density of GFAP differs between brain regions; however, our results suggest that the differences observed in the distribution of processes of astrocytes are part of the response to ischemic injury induced by 2VO and improved by physical exercise. Substantial increases in astroglial metabolism and synaptic protein synthesis, consistent with healthy cellular hypertrophy in response to increased physiological demands, indicates the importance of these cells in neuronal plasticity and regulation of cerebral microcirculation [65, 66].

Our results are in accordance with this data, as the increase in astrocytic processes found in the brains of the exercised animals was accompanied by a reduction in microglia activation, an increase in the synaptic proteins, synaptophysin and PSD-95, normalization of brain microcirculation and improvement in cognitive function.

Limitations of this study should be considered. It is well known that differences in brain regions, such as cortex and hippocampus, as well as variations in behavior between males and females. Therefore, in this study we used only male rats to avoid any hormonal interference that may occur in the results [67, 68]. However, most studies are moving away from this paradigm and in our future studies, we will make efforts to perform on heterogeneous samples that comprise male and female animals.

In conclusion, to our knowledge, this is the first study that has examined the effect of physical exercise on a chronic cerebral hypoperfusion model, reporting differences in the astrocytic coverage of cerebral blood vessels associated with microcirculation and cognitive function.

## Conclusions

Cerebral microcirculatory and inflammatory changes important in the neurodegenerative process are triggered in the initial phase of cerebral hypoperfusion and maintained during the chronic phase. We suggest that early and long-term moderate exercise induce significant improvements in brain inflammation and cognition, highlighting the neuroprotective potential of physical exercise on astrocytes coverage on brain microcirculation.

The present findings have important implications for improving care of neurodegenerative diseases. Therefore, further evaluation of physical exercise as a non-pharmacological approach may assist in the discovery of neuroprotective mechanisms. Recommending physical exercise could prevent or reduce cognitive changes and their evolution to dementia, thus improving the quality of life of these patients.

## Data Availability

The datasets used and/or analyzed during the current study are available from the corresponding author on reasonable request.

## Abbreviations

BBB: Blood-brain barrier
BSA: Bovine serum albumin
CBF: Cerebral blood flow
2VO: 2-vessel occlusion
Iba-1: Ionized calcium-binding adaptor-1
GFAP: Glial fibrillary acidic protein
MET: Maximal exercise testing
SBP: Systolic blood pressure
PSD-95: Postsynaptic density protein 95
