# Correction: Physical exercise promotes astrocyte coverage of microvessels in a model of chronic cerebral hypoperfusion

**DOI:** 10.1186/s12974-024-03138-z

**Published:** 2024-06-20

**Authors:** Marina Leardini-Tristão, Giulia Andrade, Celina Garcia, Patrícia A. Reis, Millena Lourenço, Emilio T. S. Moreira, Flavia R. S. Lima, Hugo C. Castro-Faria-Neto, Eduardo Tibirica, Vanessa Estato

**Affiliations:** 1 Laboratory of Immunopharmacology, Oswaldo Cruz Foundation, Av. Brasil, 4365, Manguinhos, Rio de Janeiro, 21040-900 Brazil; 2 Laboratory of Cardiovascular Investigation, Oswaldo Cruz Foundation, Rio de Janeiro, Brazil; 3 Laboratory of Glial Cell Biology, Biomedical Sciences Institute, Federal University of Rio de Janeiro, Rio de Janeiro, Brazil; 4 National Institute of Cardiology, Rio de Janeiro, Brazil

**Correction: Journal of Neuroinflammation (2020) 17:117** 10.1186/s12974-020-01771-y

In this article [1], the wrong figure appeared as Fig. 2; the Fig. 2 should have appeared as shown in this correction.

**Fig. 2:**
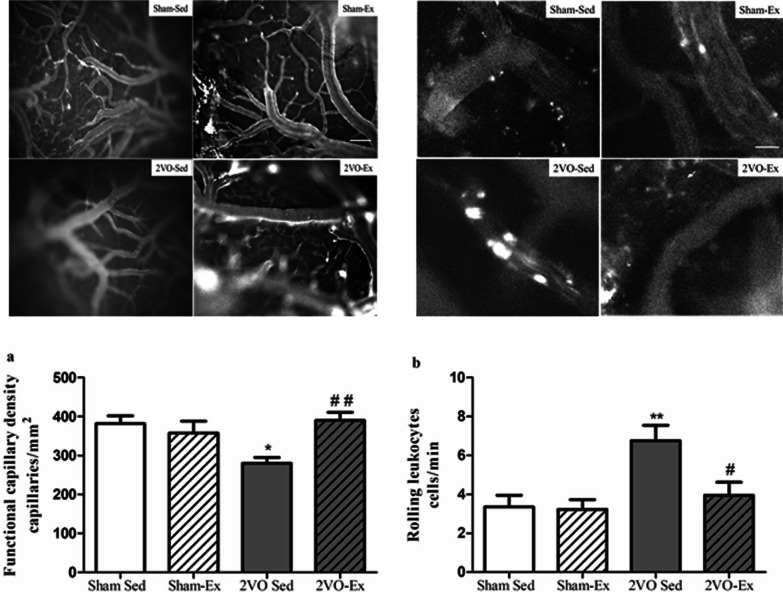
Representative images of cerebral intravital microscopy and microcirculation analysis of the cerebral cortex. The values represent the mean ± S.E.M (*n* = 6–8 per group). Bar graphs represent **a** the functional capillary density, and **b** the number of rolling leukocytes in venules after 12 weeks of physical exercise or sedentarism. Sham-Sed, sham surgery non-exercised group; Sham-Ex, sham surgery exercised group; 2VO-Sed, chronic cerebral hypoperfusion non-exercised group; 2VO-Ex, chronic cerebral hypoperfusion exercised group. In **a** *p < 0.05 vs. Sham-Sed and ^##^*p* < 0.01 vs. 2VO-Sed (unpaired Student’s t test); in **b** ^**^p < 0.01 vs. Sham-Sed and ^#^*p* < 0.05 vs. 2VO-Sed (ANOVA). Scale bar 100 μm, magnification100× in **a** and 200× in **b**
